# Post-ageing guided closed-loop discovery of multi-element alloy catalysts for automotive exhaust purification

**DOI:** 10.1039/d5na01017a

**Published:** 2026-03-16

**Authors:** Hitoshi Mikami, Azusa Kamiyama, Kohei Kusada, Megumi Mukoyoshi, Hiromasa Kaneko, Masaaki Haneda, Hiroshi Maeno, Tomokazu Yamamoto, Yasukazu Murakami, Hiroshi Kitagawa

**Affiliations:** a Honda R & D Co., Ltd, Innovative Research Excellence 4630 Shimotakanezawa, Haga-machi, Haga-gun Tochigi Japan hitoshi_mikami@jp.honda; b Kyoto University, Division of Chemistry, Kitashirakawa-Oiwakecho Sakyo-ku Kyoto 606-8502 Japan kitagawa@kuchem.kyoto-u.ac.jp; c Kyoto University, The Hakubi Center for Advanced Research, Kitashirakawa-Oiwakecho Sakyo-ku Kyoto 606-8502 Japan; d Meiji University, Department of Applied Chemistry 1-1-1 Higashi-Mita, Tama-ku Kawasaki Kanagawa Japan; e Advanced Ceramics Research Center, Nagoya Institute of Technology 10-6-29 Asahigaoka Tajimi Gifu 507-0071 Japan; f Kyushu University, The Ultramicroscopy Research Center 744 Motooka, Nishi-ku Fukuoka Japan

## Abstract

Multi-element alloy catalysts exhibit tunable electronic structures and remarkable thermal stability, making them promising materials for automotive exhaust purification. However, most data-driven explorations have emphasised fresh activity, overlooking the post-ageing durability that governs real-world performance. Here, we have developed a closed-loop high-throughput discovery framework that employs post-ageing activity as the principal design index and integrates inverse analytical prediction to accelerate the development of durable high-entropy alloy catalysts. A total of 1493 catalysts were synthesised and automatically evaluated, yielding over one hundred compositions surpassing Pd benchmarks in low-temperature activity, total conversion, and durability. Mechanistic analyses revealed that the enhanced performance originates from cooperative sites formed between different elements—indicating synergistic adsorption behaviour beyond that of individual metals—and from synthesis conditions involving low temperatures and high alkalinity, which suppress the formation of mixed oxides with alumina and thereby optimise metal–support interactions. Furthermore, multi-component evaluations including low-reactivity hydrocarbons (i-C_5_H_12_) clarified the coupled redox behaviour between NO reduction and hydrocarbon oxidation, realistically reproducing actual TWC operation. Statistical validation demonstrated over twentyfold higher discovery efficiency than random exploration (*p* < 0.001). This study establishes a durability-aware, data-driven paradigm linking alloy design, process informatics, and machine learning toward practical, platinum-group metal-efficient automotive catalysts.

## Abstract 2

A post-ageing–guided closed-loop high-throughput platform enabled the discovery of durable multi-element alloy catalysts for automotive exhaust purification through automated synthesis, durability-focused evaluation, and inverse machine learning.

## Graphical abstract

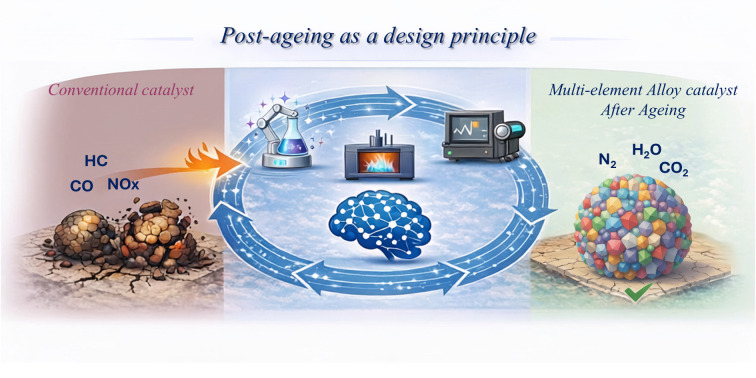

## 1. Introduction

Mobile sources such as automobiles account for a significant portion of atmospheric emissions of harmful pollutants. At the 27th Conference of the Parties to the United Nations Framework Convention on Climate Change (COP27), 41 countries agreed under the Accelerating to Zero Coalition (A2Z) framework to sell only zero-emission vehicles—electric and fuel-cell vehicles—by 2035 in leading markets and by 2040 world-wide.^1,2^

Nevertheless, the automotive industry anticipates that conventional internal-combustion-engine (ICE) vehicles will continue to occupy a non-negligible market share for the foreseeable future. Accordingly, emission regulations for gasoline and hybrid vehicles (HEVs) are expected to become increasingly stringent so as to ensure clean exhaust even under transient and low-temperature conditions.^3–5^ This tightening of global regulations has accelerated the development of advanced after-treatment technologies, including high-efficiency three-way catalysts (TWCs), electrically heated catalysts, and integrated exhaust-energy-management systems.^3^ In Europe, the transition from Euro 6 to Euro 7 represents a major milestone, imposing stricter limits on NO_*x*_, particle number, and non-methane hydrocarbons while also mandating catalyst durability throughout vehicle lifetime.^4^ Moreover, real-driving-emission (RDE) studies have revealed a degradation of gasoline-vehicle performance with increasing mileage, emphasising the need for enhanced catalyst durability and regeneration strategies.^5^ Thus, even in the era of rapid electrification, the progressive reinforcement of emission standards for hybrid and gasoline engines remains a key driving force for innovation in catalyst materials and reaction-mechanistic research.

To achieve both high catalytic activity and reduced consumption of platinum-group metals (PGMs), recent research on automotive catalysts has focused on multi-element alloy catalyst design based on the control of the electronic state of active metals.^6,7^ For instance, tuning the density of states (DOS) in Cu–Ni alloys can optimise adsorption energies and realize lower temperature oxidation of CO and hydrocarbons without using PGMs.^8^ Similarly, Pd–Cu and Pt–Cu alloys have been reported to suppress poisoning by H_2_O and NO while enabling CO and hydrocarbon oxidation at temperatures below 150 °C.^9–11^ Beyond binary and ternary alloys, multi-element alloy catalysts that exploit both electronic-structure diversity and configurational-entropy effects have emerged as a new framework capable of simultaneously achieving high activity, thermal stability, and PGM saving. A representative example is the high-entropy alloy (HEA), which contains multiple principal elements in nearly equiatomic ratios.^12–15^ HEAs possess high configurational entropy and tuneable electronic structures, providing catalytic functions that are unobtainable with conventional alloys. However, in practical synthesis, many multi-element catalysts exhibit partial segregation or local structural heterogeneity rather than complete solid-solution HEA phases, yet such materials still show excellent catalytic performance arising from entropy-driven stabilisation and electronic-structure diversity.^16,17^ Accordingly, the catalysts targeted in this study are positioned as multi-element alloy catalysts designed under the HEA concept rather than ideal single-phase HEAs.

The practical implementation of such multi-element catalysts requires precise and reproducible synthesis techniques. Recent progress in flow-type continuous synthesis systems has enabled homogeneous composition control and high-throughput screening (HTS) of multi-element catalysts.^7,18,19^ These highly flexible synthesis approaches are essential for exploring the vast compositional space of alloy catalysts and expanding the conceptual boundaries of catalyst development.

Multi-element alloy catalysts which combine high thermal stability with high catalytic activity are promising candidates for the next-generation automotive emission-control systems. The rapid evolution of HTS and machine-learning technologies now allows efficient exploration of enormous compositional spaces, marking a transition from empirical trial-and-error methods to data-driven design.^20,21^ Numerous data-driven studies have reported multi-element catalysts exhibiting high activity for CO oxidation and NO reduction;^22^ however, most of these investigations were conducted under simplified laboratory conditions, and studies addressing realistic multicomponent reaction environments remain limited.^23^ To fully exploit the potential of multi-element alloy catalysts for automotive applications, it is therefore indispensable to establish a closed-loop exploration framework that integrates HTS, machine-learning-based prediction, and experimental feedback.^24^

Previous HTS studies have predominantly focused on initial (fresh) activity, whereas evaluations of durability, sintering suppression, and post-ageing performance, which are critical for practical use, have been insufficient.^22,23^ Few studies have incorporated post-ageing conversion efficiency as a quantitative design parameter, and integration of such data with HTS or data-science analysis remains scarce. In actual emission-control catalysts, practical performance is ultimately determined not by initial activity but by long-term conversion efficiency after thermal ageing.^25^ Unlike conventional process catalysts, automotive three-way catalysts are exposed to highly dynamic and severe conditions during real driving. In industrial processes, the reaction atmosphere and temperature are typically constant, leading to predictable deactivation patterns. In contrast, automotive catalysts undergo frequent transitions between oxidising and reducing environments due to air–fuel (A/F) ratio fluctuations and fuel-cut events. These are accompanied by rapid temperature excursions often exceeding 1000 °C at the catalyst surface. These transient and cyclic conditions impose repeated redox and thermal stresses, promoting metal sintering, phase segregation, and complex structural reconstruction. Such unique operational stresses make the durability requirements for automotive catalysts far more demanding and mechanistically diverse than those for stationary process catalysts. Therefore, post-ageing activity, which reflects the reconstructed catalytic state after exposure to realistic thermal and redox stresses, represents a more relevant design index than initial activity for the discovery of practical automotive emission-control catalysts.

Moreover, recent nanoscale analyses have revealed dynamic structural rearrangements in multi-element catalysts, including HEAs, under operating conditions.^26^ These findings emphasise the necessity of a predictive closed-loop framework that optimises catalytic activity and stability simultaneously. Accordingly, the establishment of a closed-loop exploration strategy guided by post-ageing activity is expected to represent the next breakthrough toward practical multi-element alloy catalyst development.

In this work, a closed-loop exploration cycle for multi-element alloy catalysts was developed using post-ageing conversion efficiency as the principal performance index. The proposed framework features; (i) simultaneous optimisation of catalytic performance to achieve high conversion of all target gases (NO, CO, and hydrocarbons), and (ii) efficient feedback through prediction- and inverse-analysis-based approaches. This strategy aims to minimise late-stage attrition observed in conventional fresh-activity-based screening and to reduce the number of experimental iterations and total development time. The present study establishes a new research paradigm that integrates durability science, data science, and multifunctional alloy-catalyst design.

## 2. Experimental

### 2.1. Overview of the closed-loop platform

**Fig. 1:**
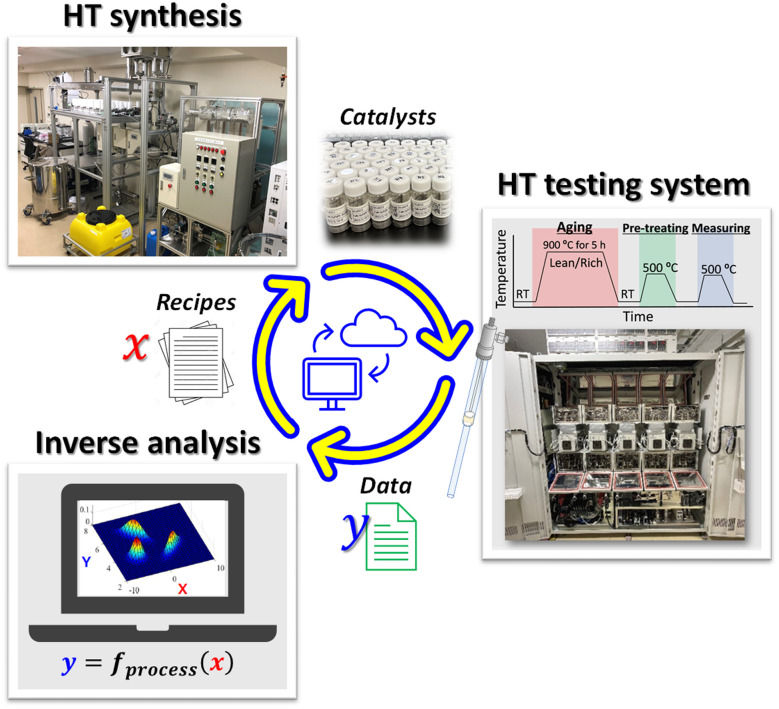
Closed loop exploration cycle consisting of a custom-built automated system based on a continuous solvothermal flow reactor, HT screening instrument for performance evaluation and inverse analysis.

To accelerate the discovery of HEA catalysts for automotive exhaust purification, a closed-loop exploration platform was constructed, as illustrated in Fig. 1. This platform integrates high-throughput (HT) catalyst synthesis, post-ageing activity evaluation using an HT testing system, and machine-learning-based direct inverse analysis to form a self-improving exploration cycle that continuously enhances both accuracy and performance. As the principal performance index, the post-ageing activity was adopted to suppress late-stage attrition caused by durability degradation and to design catalysts that retain high performance after ageing. Here, post-ageing activity was defined as the catalytic performance measured after accelerated ageing at 900 °C for 5 h under cyclic lean/rich conditions and evaluated using the temperatures required to reach 50% and 90% total conversion (*T*_50_ and *T*_90_), as well as the conversion at 350 °C under a realistic multi-component exhaust gas mixture.

The objective variables were defined as the temperatures at which the total conversion of all target gases reached 50% (*T*_50_, representing low-temperature activity) and 90% (*T*_90_, representing high-conversion performance). The target gases included NO, CO, propylene (C_3_H_6_), and isopentane (i-C_5_H_12_), representing typical oxidising and reducing components in automotive exhaust. The explanatory variables comprised the type and composition of the constituent elements, as well as the synthesis parameters such as temperature, pH, and flow rates.

This formulation enabled the simultaneous optimisation of catalytic performance to achieve a high conversion rate of all target gases (NO, CO, and hydrocarbons) under realistic multicomponent exhaust conditions.

A total of 1548 HEA catalysts were synthesised, of which 1493 were successfully evaluated. This difference mainly arises from the occasional synthesis or handling error, as well as intermittent interruptions to the automated evaluation instrument. Because the closed-loop workflow integrates synthesis, evaluation, and machine-learning prediction in iterative cycles, evaluation was intentionally paused during certain cycles when the prediction module had already generated the next experimental batch. Consequently, a subset of synthesised samples (hts1494–hts1548) was not subjected to post-ageing testing. The compositional space was systematically expanded from five-element systems to nineteen-element systems. For selected samples, both fresh and post-ageing activities were measured to analyse the effects of durability degradation quantitatively. To quantify the improvement achieved through iterative closed-loop operation, the hit ratio (*H*) relative to the Pd benchmark was defined as the fraction of samples meeting at least one of the following criteria: (1) the total conversion of NO + CO + HC exceeded that of Pd by ≥+2%, (2) *T*_50_ decreased by ≥5 °C, or (3) the conversion at 350 °C exceeded that of Pd by ≥+2%.*H* = (*N*_Pd-exceeding_/*N*_total_) × 100 (%)

This metric has enabled quantitative evaluation of the discovery efficiency achieved through iterative closed-loop exploration.

### 2.2. HT catalyst synthesis

The catalysts were synthesised using a custom-built automated system^19^ based on a continuous solvothermal flow reactor.^7^ This system can synthesise up to 20 different catalysts in a single run according to the programmed synthesis parameters, including the type and amount of metal precursor solutions, temperature (200–350 °C), pressure (20–35 MPa), and the flow rates of reductant (25% EtOH aq.; 50–100 mL min^−1^), pH-modifier (25 M NaOH aq.; 0–10 mL min^−1^), and mixed-metal precursor (10–50 mL min^−1^) solutions. All the synthesis parameters were determined through inverse analysis, except for the initial several runs, in which the parameters were randomly selected based on an experimental design approach with random numbers and D-optimality to effectively initiate the machine-learning analysis. The metal precursors (CrCl_3_·6H_2_O, MnCl_2_·4H_2_O, FeCl_2_, CoCl_2_, NiCl_2_, CuCl_2_·2H_2_O, ZnCl_2_, Na_2_MoO_4_·2H_2_O, RuCl_3_*n*H_2_O, RhCl_3_·3H_2_O, K_2_PdCl_4_, Na_2_WO_4_·2H_2_O, NaReO_4_, H_2_IrCl_6_*n*H_2_O, K_2_PtCl_4_, HAuCl_4_·3H_2_O, GaCl_3_, InCl_3_·4H_2_O, and SnCl_2_) were dissolved in 0.1 M HCl aq. at concentrations from 0.1 to 0.5 M, depending on their solubility, and then loaded into the system. The designated precursors were dispensed by a robotic pipetting arm and, after being mixed with diluted 0.1 M HCl solution, loaded into a high-pressure syringe pump. The mixed metal solution was then injected into the preheated and pH-controlled reductant stream at the designated pressure. The reaction was completed instantaneously, after which the product solution was quenched through the chiller and discharged from the outlet into a bottle containing La-doped γ-Al_2_O_3_ (SCFa-140 L3, Sasol, Germany) support under stirring. The products were filtered, rinsed with deionised water, and then dried under vacuum overnight. The total metal loading was adjusted to 1 wt% of the catalyst, and 2.5 g of each catalyst was synthesised.

### 2.3. Post-ageing activity evaluation using a HT testing system

The obtained catalysts were evaluated in three steps; ageing, pre-treatment and performance test using an HT system shown in Fig. S1(a). This instrument consists of five units, each capable of ageing, pre-treatment and activity evaluation of five samples. This configuration allows for the automatic performance evaluation of up to 25 catalysts. This enables 75 catalysts data sets to be obtained per week. Fig. S1(b) shows one unit with five fixed-bed reactors. Data for each sample were collected by switching with a revolver mechanism. The temperature was monitored with a thermocouple inserted near the top of the catalyst. To allow sequential operation of each unit, the instrument was operated using a program shown in Fig. S1(c). Initially, 100 mg of the sample was placed between two glass wool layers in a quartz tube reactor (*Φ* = 10 mm). The samples were heat-treated at 900 °C for 5 hours under model gas of switching gas of lean (O_2_, 10% H_2_O, N_2_ balance) and rich (O_2_, C_3_O_6_, 10% H_2_O, and N_2_ balance) atmosphere for degradation. The aged sample was heated to 500 °C and oxidised for 15 min under 5% O_2_, and then reduced for 15 min under 5% H_2_. Subsequently, the catalysts were heated from 100 °C to 500 °C with the ramping rate of 10 °C min^−1^ under the feed gas consisting of 500 ppm NO, 5000 ppm CO, 200 ppm C_3_H_6_, 120 ppm i-C_5_H_12_, 14% CO_2_, 4900 ppm O_2_, 1700 ppm H_2_ and N_2_ balance with a total flow rate of 500 ml min^−1^. Here, H_2_ was introduced to adjust the overall gas composition to near-stoichiometric conditions, enabling consistent relative evaluation of catalyst performance under a balanced redox environment. In addition to propylene (C_3_H_6_), isopentane (i-C_5_H_12_) was intentionally included in the feed to represent paraffinic hydrocarbons that dominate real exhaust emissions and are known to be far more resistant to oxidation under practical operating conditions.^27–29^ Because i-C_5_H_12_ oxidation requires a locally reduced surface environment similar to NO reduction, its inclusion enables direct evaluation of redox balance and coupled NO–hydrocarbon reaction behaviour under realistic three-way-catalyst conditions. The outlet gas was analysed using an FT-IR (BEST INSTRUMENTS CO., Ltd, Bex-1000FT) and a gas analyser (BEST INSTRUMENTS CO., Ltd, Bex-520 M). The conversion of NO, CO, C_3_H_6_ and i-C_5_H_12_ was calculated by (*C*_1_ − *C*_2_)/(*C*_1_ × 100%), where *C*_1_ is the gas concentration at 100 °C (0% gas conversion) and *C*_2_ refers to the gas concentration at that temperature.

### 2.4. Catalyst characterisation

FT-IR spectra of adsorbed CO species were taken with a NICOLET iS50 FT-IR spectrometer at a resolution of 4 cm^−1^ under a static condition. Prior to each experiment, a self-supporting sample disk (φ 7 mm, 12 mg) was placed in an IR cell with CaF_2_ windows. Prior to CO adsorption, the sample was subjected to the following pre-treatment sequence at 500 °C: oxidation in 66.66 kPa of O_2_, evacuation, reduction in 66.66 kPa of H_2_, and final evacuation. After pre-treatment, the cell was cooled to room temperature, and a background spectrum of the clean surface was collected for spectral correction. CO adsorption was then performed by introducing 0.40 kPa of CO at room temperature under static conditions, and spectra of the adsorbed species were subsequently recorded.

The microstructures of the catalysts were examined by high-angle annular dark-field scanning transmission electron microscopy (HAADF-STEM) using 200-kV microscopes, JEM-ARM200CF and JEM-ARM200F. The chemical compositions of the alloy nanoparticles were analysed by energy-dispersive X-ray spectroscopy (EDS) with the aforementioned electron microscopes.

### 2.5. Machine-learning-based direct inverse analysis

Metal elements, their compositions, and process conditions in catalyst synthesis were used as explanatory variables *x*, the conversions of the four chemical species were objective variables *y*, and we constructed a machine-learning model *y* = *f*(*x*) with existing experimental data. By performing inverse analysis of the constructed model, we predicted the next candidates of metal elements, their compositions, and process conditions for synthesising catalysts capable of achieving all target values of *y*. For the metal elements and their compositions of the catalysts, XenonPy^30^ was used to compute the features of the metal elements, and weighted average, weighted variance, geometric mean, harmonic mean, max-pooling, and min-pooling were calculated with the compositions, which were combined with process conditions as *x*. *y* was the conversion rate for each chemical species, derived from the experimental data.

For the inverse analysis of the model, Bayesian optimisation,^31,32^ which can explore extrapolation regions of a data set, and direct inverse analysis of the model,^32,33^ which can directly predict *x* values from target *y* values, were employed to ensure diversity of *x* candidates. In the Bayesian optimisation, a Gaussian process regression model was constructed where the kernel type was handled as a discrete hyperparameter and selected the kernel function yielding the best performance of 5-fold cross-validation from 11 kernel types. As the acquisition function, the probability of target range^34^ was calculated for each *y* and multiplied these probabilities to obtain the overall probability that all four *y* variables could achieve the target values. A genetic algorithm^35^ was used to search for metal elements, their compositions, and process conditions that maximised the acquisition function.

The model for direct inverse analysis was constructed using Gaussian mixture regression.^33^ The hyperparameters of the underlying Gaussian mixture model—namely, (i) the number of Gaussian components and (ii) the covariance type—were optimized by grid search under 5-fold cross-validation. Inputting the target values for the four *y* variables yielded the probability density function for *x*. A genetic algorithm^35^ was then used to search for metal elements, their compositions, and process conditions that maximised the probability density function.^36^ In addition, the closed-loop dataset allows an interpretable discussion of performance-driving factors by inspecting catalysts with superior performance. For example, by reviewing the compositions and synthesis conditions of high-performing catalysts, one can discuss which elements tend to be enriched and what synthesis-parameter regimes are characteristic of favourable catalytic behaviour.

## 3. Results and discussion, experimental

### 3.1 Closed-loop discovery guided by post-ageing activity

We implemented a closed-loop exploration strategy using post-ageing activity as the primary performance metric. Across 1493 synthesised and evaluated catalysts, we identified approximately 100 compositions outperforming the Pd benchmark. More specifically, we obtained 106 samples with improved low-temperature activity (Δ*T*_50_ ≤ −5 °C *vs.* Pd), 290 samples with ≥+2% gain in NO and i-C_5_H_12_ conversion at 350 °C, and 124 samples showing ≥+2% improvement for all species (NO, CO, C_3_H_6_, i-C_5_H_12_) over 150–450 °C. These results confirm that numerous multi-element alloy catalysts can surpass Pd across multiple performance indicators.

**Fig. 2:**
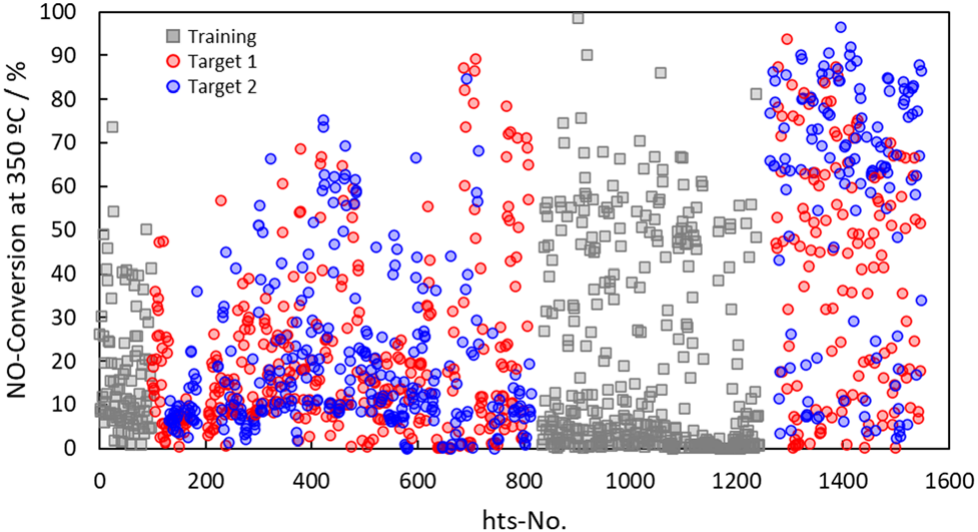
Post-ageing performance of NO conversion at 350 °C with enlarging candidate elements from 5 to 19.

Fig. 2 shows the evolution of NO conversion at 350 °C across successive exploration cycles. As the compositional design space expanded from five to nineteen elements, the activity distribution evolved from a narrow, low-activity region (<30%) in the initial phase to a broader, high-activity region in later cycles. This progressive shift demonstrates that iterative learning effectively enhanced the discovery efficiency by directing exploration toward high-performance regions.

The key factors enabling this efficient discovery can be organised along two complementary axes: (A) real-world applicability – prioritising post-ageing activity over initial (fresh) activity to directly evaluate structural reconstruction and metal–support interactions relevant to realistic catalyst durability (discussed in Sections 3.2–3.4). (B) Data-driven efficiency optimisation – integrating machine learning, inverse analysis, and experimental feedback to converge both elemental selection and synthesis parameters toward high-activity regions, achieving statistically significant improvements in discovery efficiency (validated in Section 3.5).

In the following sections, we examined; (i) the mechanistic insights derived from IR spectroscopy regarding post-ageing activity changes (*e.g.*, Cu–Pt cooperative site formation and Pt diffusion deactivation); (ii) the role of synthesis process optimisation, particularly under low-temperature and high-alkali conditions; (iii) the multi-indicator optimisation approach linking NO–i-C_5_H_12_ conversions and oxygen balance; and (iv) the statistical validation of efficiency enhancement *via* predictive, inverse-analytical closed-loop learning. This comprehensive framework demonstrates the synergy among durability-oriented design, data-driven exploration, and process optimisation in accelerating catalyst discovery.

### 3.2 Real-world applicability: effect of post-ageing–based HT exploration

Conventional catalyst screening based on fresh activity often fails to account for degradation effects, leading to late-stage attrition of promising candidates. To address this limitation, we evaluated the catalytic activities of 508 samples before and after an accelerated ageing process to quantitatively assess structural and performance evolution. As a result, more than 99% of the catalysts exhibited measurable changes in at least one activity index after ageing, indicating that thermal ageing induces substantial functional reconstruction and supporting the validity of adopting post-ageing activity as the primary design index.

Table 1 summarises the statistical distribution of post-ageing effects across three key activity indices: (i) *T*_50_ (temperature for 50% total conversion), (ii) conversion at 350 °C, and (iii) overall total conversion (150–450 °C). In this analysis, the categories of “total decrease”, “stable”, and “total improvement” denote samples in which the activities of all four gases (NO, CO, C_3_H_6_, and i-C_5_H_12_) simultaneously decreased, remained unchanged, or increased, respectively. In contrast, “mixed behaviour” represents the remaining fraction of samples exhibiting non-uniform trends—namely, those showing improvement in some gases while deterioration in others. More than 99% of the tested catalysts exhibited measurable changes in at least one index, confirming that ageing is not a simple stabilisation step but a dynamic restructuring process that alters catalytic behaviour in nearly every case. Only a negligible fraction (<1%) maintained constant activity across all gases, whereas the vast majority showed mixed behaviour, highlighting the complex interplay between metal dispersion, alloy reconstruction, and support interaction. The coexistence of total and mixed trends implies that post-ageing performance is determined by a delicate balance between reconstruction-driven activation and sintering-induced deactivation, underscoring the highly dynamic nature of HEA catalyst systems under thermal stress.

**Table 1:** Statistical summary of post-ageing effects on 508 catalysts

Index	Total decrease	Stable	Total improvement	Mixed behaviour
*T*_50_	6.3	0.2	8.3	85.2
Conversion at 350 °C	11.0	0.0	21.9	67.1
Total conversion	10.0	0.4	18.5	71.1

**Fig. 3:**
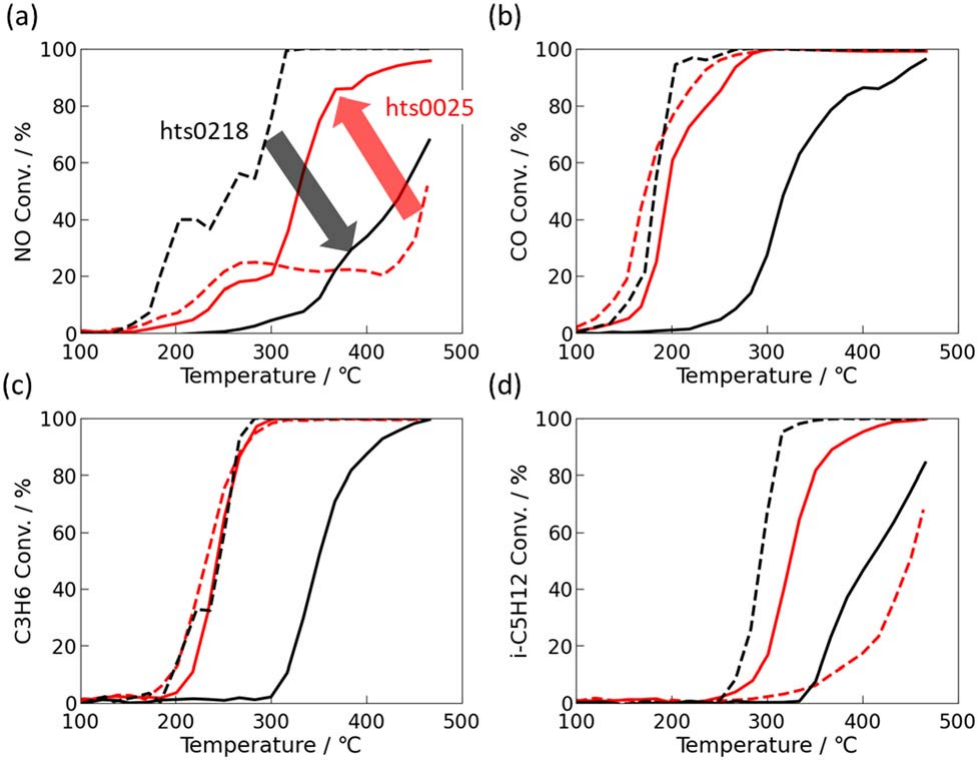
Comparison of light-off curves between fresh (solid) and aged (dashed): of FeNiCuPdPt(=19 : 17 : 11 : 27 : 27 at%)/Al_2_O_3_ (black) and FeNiCuPdPt(=3 : 10 : 2:6 : 79 at%)/Al_2_O_3_ (red): (a) NO, (b) CO, (c) C_3_H_6_ and (d) i-C_5_H_12_.

Fig. 3 shows two representative cases; hts0218 (degraded after ageing) and hts0025 (improved after ageing). Their nominal compositions are summarised in Table S1. hts0218 exhibited a significant loss of activity after ageing. The *T*_50_ values for NO, CO, C_3_H_6_, and i-C_5_H_12_ shifted to higher temperatures by 180, 147, 105, and 136 °C, respectively, and the conversions at 350 °C decreased by 88, 29, 49, and 92%. In contrast, hts0025 showed improved post-ageing performance: the *T*_50_ values decreased by 134 °C (NO) and 124 °C (i-C_5_H_12_), while remaining nearly unchanged for CO and C_3_H_6_, which were already fully converted (100%). The 350 °C conversions increased by 52% (NO) and 75% (i-C_5_H_12_), again confirming the enhanced low-temperature activity and overall robustness.

The upper panels in Fig. S2 present high-angle annular dark-field scanning transmission electron microscopy (HAADF-STEM) images of hts0218 in the states (a) before and (b) after ageing. The bright regions indicated by yellow arrows correspond to metallic particles supported on Al_2_O_3_. The catalyst in (a) exhibits agglomerates composed of particles approximately 20 nm or smaller, whereas the catalyst in (b) contains large particles exceeding 50 nm in size. The lower panels in Fig. S2 show another set of HAADF-STEM images for hts0025 (c) before and (d) after ageing. Similar to the observations for hts0218, the catalyst before ageing consists of agglomerates of fine particles, while the specimen after ageing exhibits much larger particles exceeding 50 nm in size. Thus, for the catalysts before ageing, HAADF-STEM observations revealed no significant changes in the dispersion or size of the metallic nanoparticles. Likewise, the catalysts after ageing exhibited no appreciable changes in dispersion or particle size. These results indicate that the degradation and improvement behaviours observed after ageing cannot be attributed to morphological changes in the catalysts.

**Fig. 4:**
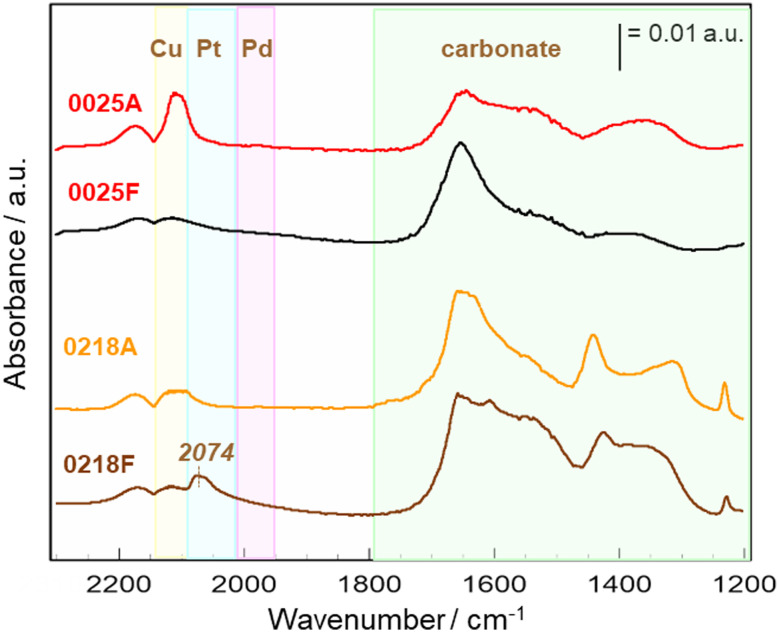
CO-adsorption DRIFTS spectra of hts0218 and hts0025 before (F) and after (A).

CO-adsorption IR spectroscopy (Fig. 4) provided insight into the surface electronic structure. Both catalysts contained Pd, Pt, Cu, Ni, and Fe, yet their electronic responses to ageing differed markedly. For hts0218, a distinct linear Pt–CO stretching band was evident in the fresh state, confirming Pt as the dominant CO adsorption site. After ageing, this Pt–CO band almost completely disappeared, with no new bands from Pd, Cu, or Ni. Given that STEM showed no morphological changes, and that Pt does not readily form stable mixed oxides with Al_2_O_3_, this disappearance is attributed to electronic isolation of surface Pt atoms through coordination with neighbouring Ni and Fe atoms, which suppress CO adsorption strength.^37,38^ In contrast, hts0025 showed no distinct CO adsorption bands in the fresh state, implying a partially oxidised or electronically dispersed surface. After ageing, new CO bands corresponding to Cu–CO and Pt–CO species appeared, accompanied by subtle wavenumber shifts indicative of Cu–Pt electronic coupling. This suggests that thermal treatment induced surface segregation and atomic rearrangement, forming Cu–Pt cooperative sites that facilitate CO adsorption and activation.^39–42^ At the same time, the absence of strongly bound CO species on some alloy surfaces indicates weakened CO adsorption due to alloying-induced electronic modification, which may mitigate CO poisoning and contribute to improved low-temperature activity; however, a direct quantitative correlation between nanoscale segregation and catalytic performance cannot be established based solely on the present *ex situ* analyses. Such reconstruction enhanced redox buffering and oxygen mobility, leading to the improved NO and hydrocarbon conversions observed in Fig. 3.

Overall, the combined STEM and IR results demonstrate that the contrasting post-ageing behaviours of hts0218 and hts0025 originate from nanoscale electronic restructuring rather than morphological degradation. The loss of Pt–CO sites in hts0218 reflects electronic deactivation through metal–metal coordination, while the emergence of Cu–Pt cooperative sites in hts0025 represents beneficial reconstruction driven by intermetallic electron exchange. These findings emphasize that post-ageing activity in HEA catalysts is governed by element-specific electronic coupling and redistribution, not by simple sintering or oxide formation.^37–42^ Accordingly, post-ageing–based screening enables efficient selection of reconstruction-enabled, electronically adaptive, and durable catalysts that would be overlooked by conventional fresh-state screening. Optimising the interaction between noble and base metals—particularly Pt–Cu and Pt–Ni combinations—while maintaining moderate metal–support interaction is key to achieving stable dispersion and superior durability. This design principle, guided by post-ageing performance, successfully identified multiple HEA catalysts surpassing the Pd benchmark in both activity and robustness.

### 3.3 Synthesis matters: process optimisation within the closed loop

**Fig. 5:**
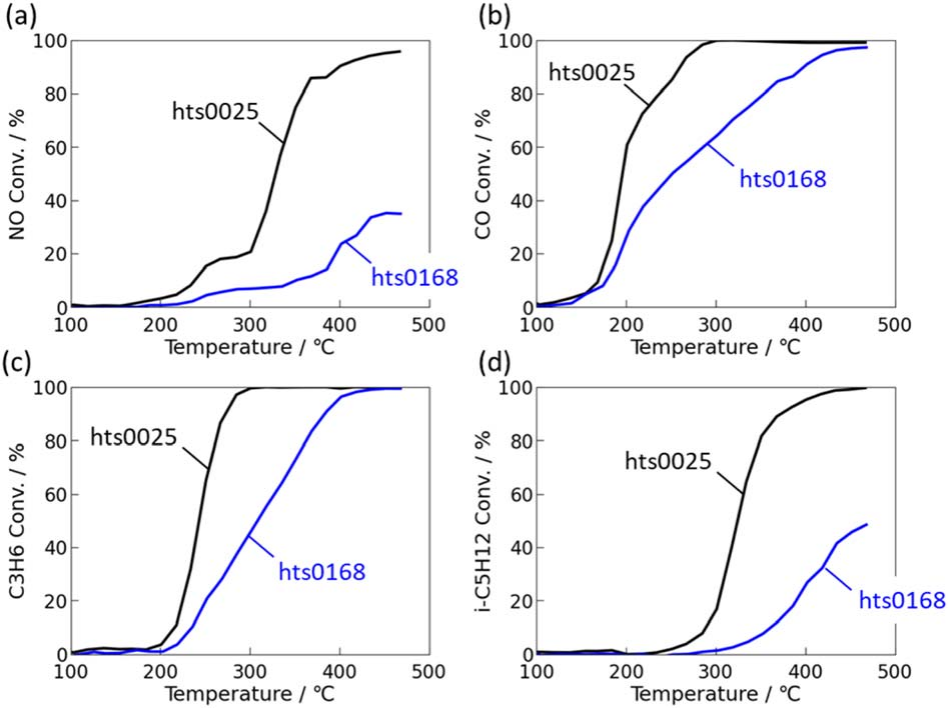
Comparison of post-ageing-light-off curves of PdFePtCuNi–Al_2_O_3_ catalysts synthesised with different process parameters: (a) NO, (b) CO, (c) C_3_H_6_ and (d) i-C_5_H_12_ conversion.

Fig. 5 compares the catalytic performance of two multi-element alloy catalysts, hts0025 and hts0168, both composed of Fe, Ni, Cu, Pd, and Pt in nearly equiatomic proportions (*ca.* 20 at% each). Despite their identical elemental compositions, their conversion behaviours differ substantially. Table 2 summarises the corresponding synthesis parameters: hts0025 was synthesised under a higher NaOH flow rate (7.4 mL min^−1^) and lower temperature (245 °C), whereas hts0168 employed a lower NaOH flow rate (4.5 mL min^−1^) and higher temperature (264 °C). hts0025 exhibited markedly higher activity across all target gases. The light-off temperature (*T*_50_) was reduced by 54 °C for CO and 71 °C for C_3_H_6_ compared with hts0168, while hts0168 failed to reach 50% conversion for NO and i-C_5_H_12_. At 350 °C, hts0025 achieved conversion improvements of +64% for NO, +21% for CO, +28% for C_3_H_6_, and +73% for i-C_5_H_12_, demonstrating superior low- and mid-temperature performance. These results confirm that the synthesis process critically governs catalytic activity, even when the nominal composition remains unchanged.

**Table 2:** Process parameters of hts0025 and hts0168

	Element type					Solvent		Reductant		pH-modifier		Temp/°C	Press/MPa
	Fe	Ni	Cu	Pd	Pt	HCl/mol L^−1^	Flow/ml min^−1^	EtOH/%	Flow/ml min^−1^	NaOH/mol L^−1^	Flow/ml min^−1^		
hts0025	19	17	11	27	27	0.1	25	25	100	25	7.4	245	25
hts0168	19	17	23	24	18	0.1	26	25	100	25	4.5	264	25

The enhanced performance under low-temperature and high-alkali-feed conditions can be rationalised by combined solution-phase and solid-state effects. An appropriate NaOH flow rate is essential: if the alkalinity is too low, premature reduction occurs before the reducing agent is introduced, leading to isolated coarse metallic particles with poor dispersion. A sufficiently high NaOH supply promotes the formation of metal–hydroxide or complex precursors, maintaining metals in ionic form until controlled reduction proceeds. This ensures synchronous multi-metal reduction and homogeneous nucleation, yielding fine, compositionally uniform alloy nanoparticles.

Reaction temperature plays a dual role. Elevated temperature accelerates reduction kinetics but also enhances atomic diffusion and particle coarsening. Hence, the optimal regime involves complete reduction of all constituent metals at the lowest feasible temperature, effectively suppressing sintering and phase segregation while preserving nano-alloy uniformity and active interfacial ensembles. These combined effects—sufficient alkalinity to stabilize precursor chemistry and moderately low temperature to limit growth—synergistically yield catalysts with stabilized microstructure, enhanced redox flexibility, and optimised electronic environments suitable for multi-gas conversion.

The microstructures of the two catalysts, hts0025 and hts0168, were examined by HAADF-STEM and EDS. The average size of the metallic nanoparticles was 53.2 nm for hts0025, which was larger than that of hts0168 (36.2 nm) (HAADF-STEM observations, not shown here). These values were obtained from measurements of 36 and 25 individual nanoparticles for hts0025 and hts0168, respectively. The chemical compositions of the nanoparticles were determined by EDS, as summarised in Fig. S3. Regarding the structure of the support in catalyst hts0025, as shown in Fig. S4(1), La–Al–based oxide was formed in the vicinity of the metallic nanoparticles. In addition, catalyst hts0025 exhibited small particles of La_2_O_3_. These compounds were also observed in catalyst hts0168, as shown in Fig. S4(2). The elemental maps of hts0168 suggest that the crystal grains of La–Al–based oxide contain other elements such as Fe, Ni, and Cu. Indeed, these oxides (La–Al–based oxide and La_2_O_3_) were observed more frequently in hts0168 than in hts0025. Thus, electron microscopy revealed distinct differences in the microstructures of the two catalysts. Importantly, La–Al–based oxides and La_2_O_3_ species were observed in both catalysts exhibiting post-ageing improvement and degradation, indicating that support evolution itself does not determine the direction of post-ageing performance.

It is likely that superior activity of hts0025 arises not only from its nominal composition but also from the microstructural and electronic states encoded by the synthesis process. For multicomponent systems such as HEAs, synthesis parameters—including pH, NaOH flow rate, temperature, reducing-agent flow, and pressure—directly control phase formation, lattice distortion, and defect chemistry.^13,43^ In our closed-loop framework, these parameters were treated as learnable variables. Through iterative feedback, the algorithm autonomously identified a process window favouring low temperature and high alkali dosage, consistently yielding catalysts with higher post-ageing activity. This trend is not a model-driven artifact but reflects an underlying experimental distribution that was statistically enriched through predictive exploration, as evidenced by the comparison between random and predictive searches (Fig. S6 and Table 3). This demonstrates that “how a catalyst is made” is as decisive as “what it is made of”. The integrated perspective—linking composition × structure × process—establishes a foundation for process-informatics–driven catalyst design.

**Table 3:** Comparison of random and predictive exploration

Metric	Random exploration	Predictive exploration	Statistical significance
Sample count (*n*)	393	382	—
*T*_50_ improvement	1.3% (95% CI: 0.3–2.6%)	27.0% (95% CI: 21.9–32.7%)	*p* < 0.001
Conversion at 350 °C	3.1% (95% CI: 1.6–5.4%)	44.3% (95% CI: 38.5–50.3%)	*p* < 0.001
Overall conversion (NO + CO + HC)	1.5% (95% CI: 0.6–3.2%)	37.2% (95% CI: 31.4–43.3%)	*p* < 0.001

### 3.4 Multi-indicator optimisation of NO, CO, and hydrocarbon conversions

**Fig. 6:**
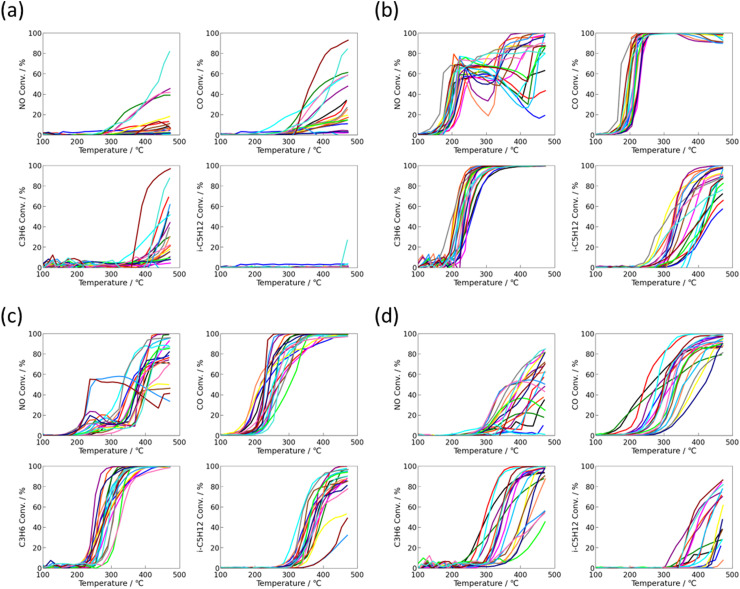
Post-ageing-light-off curves clustered by *k*-means: (a) low activity, (b) high activity, (c) low-temperature NO-deficient, and (d) CO/C_3_-selective oxidation. Each cluster exhibits distinct light-off behaviours reflecting differences in redox balance and surface oxygen dynamics. CO and C_3_H_6_ typically ignite first (200–250 °C) under oxygen-rich conditions, while NO and i-C_5_H_12_ show delayed and coupled light-off above 350 °C. The concurrent rise of NO and i-C_5_H_12_ conversions indicates dynamic redox coupling between hydrocarbon oxidation and NO reduction, where paraffin oxidation consumes surface oxygen and promotes the formation of locally reduced sites for NO activation.

Automotive three-way catalysts (TWCs) must remove CO, hydrocarbons (HCs), and nitrogen oxides (NO_*x*_) from gasoline exhaust simultaneously. Achieving high conversion for a single component is insufficient because these reactions are mutually coupled through the catalyst's redox state and surface oxygen coverage. Fig. 6 classifies the 1493 activity profiles into four clusters using *k*-means clustering; (a) low-activity, (b) high-activity, (c) low-temperature NO-deficient, and (d) CO/C_3_-selective oxidation types. Here, *k*-means was employed as an exploratory visualization tool to identify characteristic catalytic behaviors rather than for strict classification. The number of clusters (*k* = 4) was selected based on the elbow method, which shows a clear inflection at *k* = 4 (Fig. S7), and represents the minimal number of clusters that yields physically interpretable groups with distinct redox and oxidation–reduction characteristics. CO and C_3_H_6_ typically ignite first (at 200–250 °C) and reach high conversion under oxygen-rich conditions, whereas NO and i-C_5_H_12_ show delayed light-off (>350 °C) and their conversions increase in a coupled manner.

Fig. S5 shows the Pd catalyst light-off together with O_2_, NO, NO_2_, N_2_O, NH_3_ concentration profiles. At low-temperature (*ca.* 300 °C) where NO and i-C_5_H_12_ do not yet ignite, O_2_ remains above 1000 ppm, *i.e.*, the gas phase is excess-oxidising. Because i-C_5_H_12_ is unreactive there, O_2_ is not consumed and NO reduction is suppressed, favouring oxidative pathways (N O_2_/N_2_O formation). As temperature rises and i-C_5_H_12_ oxidation accelerates, O_2_ drops sharply (*ca.* 350 °C), and NO reduction turns on abruptly. Hence, O_2_ depletion *via* i-C_5_H_12_ oxidation and NO reduction are interlocked, consistent with TWC fundamentals; CO/C_3_H_6_ oxidation proceeds under oxygen-rich conditions, whereas NO reduction and i-C_5_H_12_ oxidation require a more reducing environment. The temperature window where NO and i-C_5_H_12_ light off together thus reflects an optimised redox balance.

Accounting for this interdependence clarifies the value of multi-indicator optimisation. Although absolute NO reduction levels may depend on H_2_ concentration, the relative performance ranking under identical near-stoichiometric feed conditions is primarily governed by the overall redox balance involving hydrocarbon oxidation, oxygen consumption, and surface redox capability, and is therefore expected to be robust against moderate variations in H_2_ concentration. Single-component targeting tends to bias either oxidation or reduction. Co-optimising NO/CO/HC conversion enables coordinated control of oxygen mobility, surface electron donation, and redox environment. In the loop, we used low-temperature (*T*_50_) and mid-temperature (350 °C) targets and explicitly included NO–i-C_5_H_12_ coupling descriptors, balancing kinetic and thermodynamic factors. *k*-means clustering helped visualize the multidimensional landscape and quantify relationships among reaction pathways.

This coupling can be rationalised by the fundamental mechanism of TWC reactions; CO and light-hydrocarbon (C_3_H_6_) oxidation proceed predominantly under oxygen-rich conditions, whereas NO reduction and heavy-hydrocarbon (i-C_5_H_12_) oxidation favour reducing environments.^44,45^ Hence, the simultaneous rise in NO and i-C_5_H_12_ conversions marks the critical redox balance point in the catalytic system. Including such low-reactivity paraffinic hydrocarbons in the test composition thus provides a realistic approximation of gasoline exhaust conditions, revealing the competitive and complementary nature of oxidation and reduction pathways in practical operation. Here, i-C_5_H_12_ was intentionally selected as a low-reactivity paraffinic hydrocarbon to serve as a stringent probe for sensitively discriminating redox balance and reaction coupling under multi-component conditions, rather than to fully replicate real exhaust compositions.

Overall, multi-objective optimisation that considers residual O_2_, HC oxidizability, and NO reducibility is essential under realistic exhaust conditions and forms a core element of the closed-loop strategy.

### 3.5 Efficiency gains from predictive and inverse-analytical closed-loop learning

Table 3 summarises the statistical comparison between random and predictive exploration. The predictive method achieved markedly higher hit rates for all three activity indices: *T*_50_ improvement (≥5 °C *vs.* Pd), conversion at 350 °C (≥+ 2% *vs.* Pd), and total conversion over 150–450 °C (≥ +2% *vs.* Pd). These values were 27.0%, 44.3%, and 37.2%, respectively, compared to 1.3%, 3.1%, and 1.5% for random exploration. The 95% confidence interval (95% CI; confidence interval) indicates the statistical range within which the true hit rate is expected to fall with 95% probability; in all cases, the intervals for random and predictive exploration did not overlap, confirming a statistically significant difference (*p* < 0.001, Fisher's exact test). Overall, this equates to an increase in discovery efficiency of 20–30 times, demonstrating that model-guided exploration effectively focuses experimental efforts on high-performance regions within the compositional space. A summary of the top 15 multi-element alloy catalysts ranked by post-ageing total conversion (150–450 °C) is provided in Table S2. The table lists the rank, sample ID, constituent elements, nominal compositions (at%), total conversion integrated over the temperature range of 150–450 °C after ageing, and the relative total conversion expressed as a fold improvement over the Pd benchmark (×Pd), where the relative total conversion is defined as the ratio of the post-ageing total conversion of each catalyst to that of the Pd reference measured under identical evaluation conditions.

Fig. S6(a) illustrates the compositional bias underlying this improvement. Random exploration sampled all 19 elements nearly uniformly, while predictive exploration concentrated on Pd, Rh, and Ga and reduced the selection of Ni and Co, indicating that the model learned to favour combinations correlated with high post-ageing activity. This selection frequency provides a simple proxy for feature importance, suggesting that Pd and Rh act as dominant contributors to intrinsic catalytic activity, whereas Ga plays a supporting role in modulating post-ageing stability and reconstruction behaviour. Fig. S6(b) highlights the most distinctive process parameters—namely, higher alkali dosage and slightly lower synthesis temperature—where predictive exploration deviated strongly from random sampling. Together with the elemental trends in Fig. S6(a), these results indicate that both elemental selection and synthesis parameters contribute cooperatively to achieving high post-ageing performance. These trends are consistent with the observations in Section 3.3, where catalysts synthesised under low-temperature and high-alkali conditions exhibited superior durability and redox balance. Importantly, the improvement in hit rate achieved by predictive exploration is statistically supported by non-overlapping 95% confidence intervals and Fisher's exact test, indicating that the enrichment of the high-alkalinity and low-temperature synthesis window reflects a genuine experimental trend rather than a model-driven bias (Table 3).

**Fig. 7:**
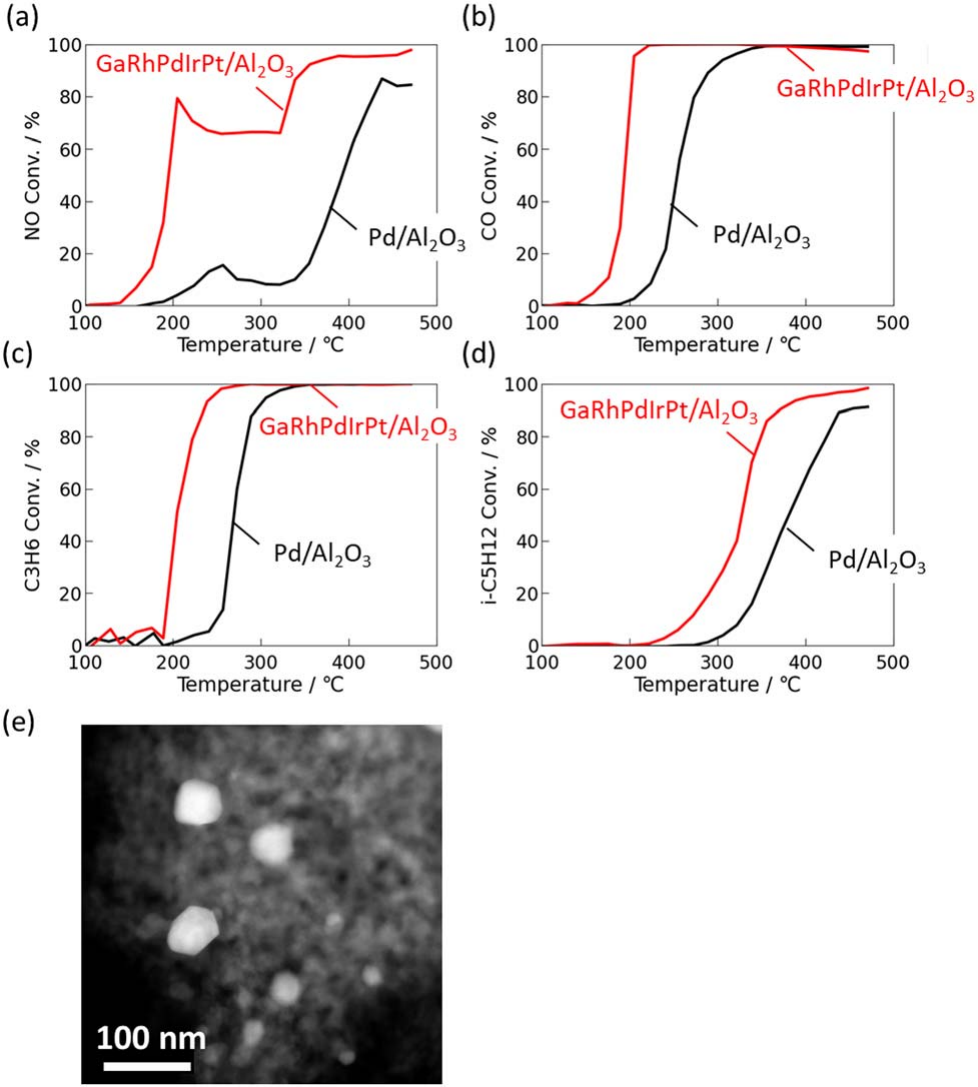
Light-off curves of GaRhPdIrPt-Al_2_O_3_ catalysts synthesised with different process parameters: (a) NO, (b) CO, (c) C_3_H_6_ and (d) i-C_5_H_12_ conversion, (e) HAADF-STEM image.

Fig. 7 shows the light-off behaviour of a representative predictive hit, hts1321, which exhibits the characteristic features identified as conducive to high post-ageing activity. The catalyst comprises Pd, Rh, and Ga, elements frequently highlighted by the model as favourable for enhanced redox balance and cooperative noble-metal effects. It was synthesised under high alkali dosage and low synthesis temperature, conditions previously correlated with improved surface dispersion and structural stability. At 350 °C, the conversions of NO, CO, and hydrocarbons all exceeded 80%, and the NO *T*_50_ was approximately 200 °C lower than that of the Pd reference catalyst. When the HAADF-STEM image of hts1321 was examined, several metallic nanoparticles—approximately ten discernible particles within the field of view—were observed on the oxide surface, with estimated diameters in the range of 20–50 nm. Although this observation is based on a single image and thus represents a limited sampling area, no apparent agglomeration or structural irregularity was detected. While detailed microstructural analysis is beyond the scope of this study, this qualitative observation supports that the synthesised catalyst exhibits a representative nanostructure typical of the high-activity group identified through the closed-loop screening.

This outstanding light-off performance confirms that the model successfully extracted the intrinsic physicochemical descriptors—namely, noble-metal synergy, redox equilibrium, and local pH buffering—that determine post-ageing catalytic behaviour.

### 3.6 Limitations and outlook

Future studies may extend the present screening framework by incorporating additional exhaust-relevant components, such as more reactive hydrocarbons or chemically diverse species, to further bridge high-throughput catalyst discovery with real-world automotive exhaust environments.

Looking ahead, the focus of further development of the closed-loop exploration framework will be on enhancing its practicality and societal relevance. Incorporating cost efficiency and resource availability as additional objective variables, alongside catalytic performance, will enable the discovery of HEA catalysts that are optimised for activity, durability, economic sustainability and material sustainability. This approach is particularly important for reducing dependence on scarce platinum-group metals and for identifying combinations of abundant elements that are suitable for large-scale applications.

Moreover, future studies will integrate physicochemical property evaluation—including surface structure analysis, electronic state characterisation, and thermal stability assessment—into the closed-loop system. Such multi-objective optimisation will allow the algorithm to learn direct correlations between intrinsic material properties and real-world catalytic behaviour, thereby bridging the gap between computational prediction and practical catalyst design.

Ultimately, by combining performance, cost, and resource sustainability within a unified optimisation scheme, the proposed framework can evolve into a comprehensive, data-driven platform for developing next-generation HEA catalysts with both scientific significance and industrial feasibility.

## 4. Conclusions

In this work, a closed-loop discovery framework integrating post-ageing-based evaluation, multi-objective optimisation, process informatics, and inverse-analytical prediction was established for efficient exploration of multi-element alloy catalysts for automotive three-way catalysis. A total of 1493 samples were synthesised and evaluated, yielding over one hundred catalysts that outperformed Pd benchmarks in low-temperature activity, overall conversion, and durability after ageing.

From an application-oriented standpoint, four factors were identified as key to achieving practical and durable performance: (1) adopting post-ageing activity as the principal index effectively prevented regression after thermal degradation; (2) incorporating synthesis parameters clarified that process conditions strongly influence structure and performance; (3) employing realistic exhaust mixtures containing low-reactivity hydrocarbons (*e.g.*, i-C_5_H_12_) revealed the coupled redox behaviour of hydrocarbon oxidation and NO reduction; and (4) multi-objective optimisation across NO, CO, and HC conversions enabled balanced oxidation–reduction control for high total conversion.

From an efficiency-oriented perspective, the closed-loop system integrating predictive modelling and inverse analysis achieved a discovery efficiency that was more than twentyfold higher than that of random exploration, as statistically validated by Fisher's exact test (*p* < 0.001). This demonstrates that the model not only accelerates experimental screening but also autonomously learns the scientific rationale underlying high catalytic activity—such as durability, redox balance, and process-dependent structure formation—and transfers this knowledge into subsequent exploration cycles.

In conclusion, the present study establishes a new paradigm for durability-aware, data-driven catalyst discovery, bridging practical performance requirements with machine-learning-driven efficiency. This approach offers a robust foundation for industrial-scale catalyst design, and future extensions incorporating cost, resource availability, and *in situ* structural characterisation are expected to further advance toward practical implementation.

## Author contributions

H. M. and H. Kitagawa conceived the research concept, designed the experimental strategy, and led the overall project supervision. K. K. and M. M. conducted the high-throughput catalyst synthesis. A. K. performed the automated high-throughput activity evaluation and data acquisition. H. Kaneko developed and implemented the inverse analytical and machine-learning models. M. H. conducted the infrared spectroscopic measurements. H. M., T. Y., and Y. M. contributed to the STEM measurements. All authors discussed the results and contributed to writing and revising the manuscript.

## Conflicts of interest

There are no conflicts to declare.

## Supplementary Material

NA-OLF-D5NA01017A-s001

## Data Availability

All data supporting the findings of this study are available within the article and its supplementary information (SI). Additional raw datasets generated and analysed during the current study are proprietary to Honda R & D Co., Ltd, and are available from the corresponding author upon reasonable request. Supplementary information: experimental details, additional figures and tables. See DOI: https://doi.org/10.1039/d5na01017a.
