# Supplementary material for: Post-ageing guided closed-loop discovery of multi-element alloy catalysts for automotive exhaust purification
Source: Nanoscale Adv. 2026 Mar 16;8(9):2896–907. doi: 10.1039/d5na01017a (PMC12991304; doi:10.1039/d5na01017a)
Supplement: NA-008-D5NA01017A-s001 [file NA-008-D5NA01017A-s001.pdf]

Supplementary Information (SI) for Nanoscale Advances.
This journal is © The Royal Society of Chemistry 2026

Supporting Information

Post-aging guided closed-loop discovery of multi-element alloy catalysts for automotive exhaust purification

Hitoshi Mikami,[a] Azusa Kamiyama,[a] Kohei Kusada,[bc] Megumi Mukoyoshi,[b] Hiromasa Kaneko,[d] Masaaki Haneda,[e] Hiroshi Maeno,[f] Tomokazu Yamamoto,[f] Yasukazu Murakami,[f] and Hiroshi Kitagawa*[b]*

[a] Honda R&D Co., Ltd., Innovative Research Excellence, 4630 Shimotakanezawa, Haga-machi, Haga-gun, Tochigi, Japan.

[b] Kyoto University, Division of Chemistry, Kitashirakawa-Oiwakecho, Sakyo-ku, Kyoto, Japan

[c] Kyoto University, The Hakubi Center for Advanced Research, Kitashirakawa-Oiwakecho, Sakyo-ku, Kyoto, Japan

[d] Meiji University, Department of Applied Chemistry, 1-1-1 Higashi-Mita, Tama-ku, Kawasaki, Kanagawa, Japan

e Advanced Ceramics Research Center, Nagoya Institute of Technology, 10-6-29 Asahigaoka, Tajimi, Gifu 507-0071, Japan

f Kyushu University, The Ultramicroscopy Research Center, 744 Motooka, Nishi-ku, Fukuoka, Japan

(a)

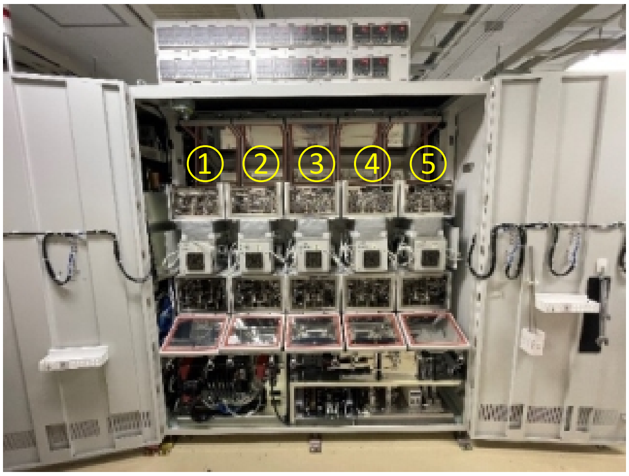

(b)

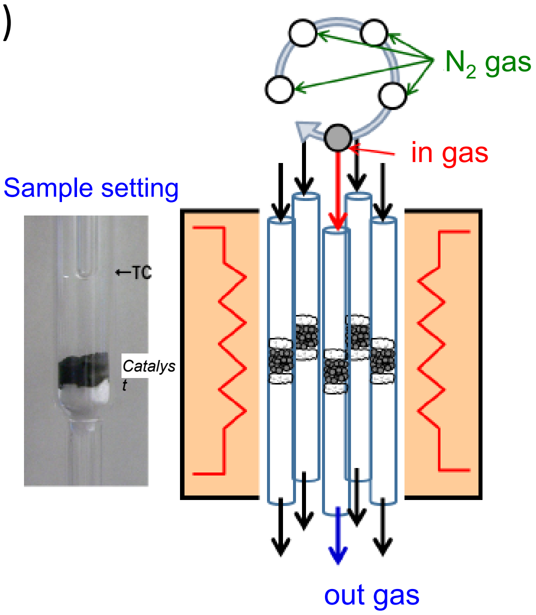

(c)

**Unit 1**	**Pre-treatment**	**Fresh-evaluation**	**Aging**	**Pre-treatment**	**Aged-evaluation**	**Cooling**				
**Unit 2**		**Pre-treatment**	**Fresh-evaluation**	**Aging**	**Pre-treatment**	**Aged-evaluation**	**Cooling**			
**Unit 3**			**Pre-treatment**	**Fresh-evaluation**	**Aging**	**Pre-treatment**	**Aged-evaluation**	**Cooling**		
**Unit 4**				**Pre-treatment**	**Fresh-evaluation**	**Aging**	**Pre-treatment**	**Aged-evaluation**	**Cooling**	
**Unit 5**					**Pre-treatment**	**Fresh-evaluation**	**Aging**	**Pre-treatment**	**Aged-evaluation**	**Cooling**

Fig. S1 High-throughput screening instrument for performance evaluation with five bed-reactors. (a) Photograph, (b) the diagram of a bed fixed reactors , (c) operation program of five unites.

Table S1 Preparation composition of hts0025 and hts0218.

	Fe	Ni	Cu	Pd	Pt
hts0025	19	17	11	27	27
hts0218	3	10	2	6	79

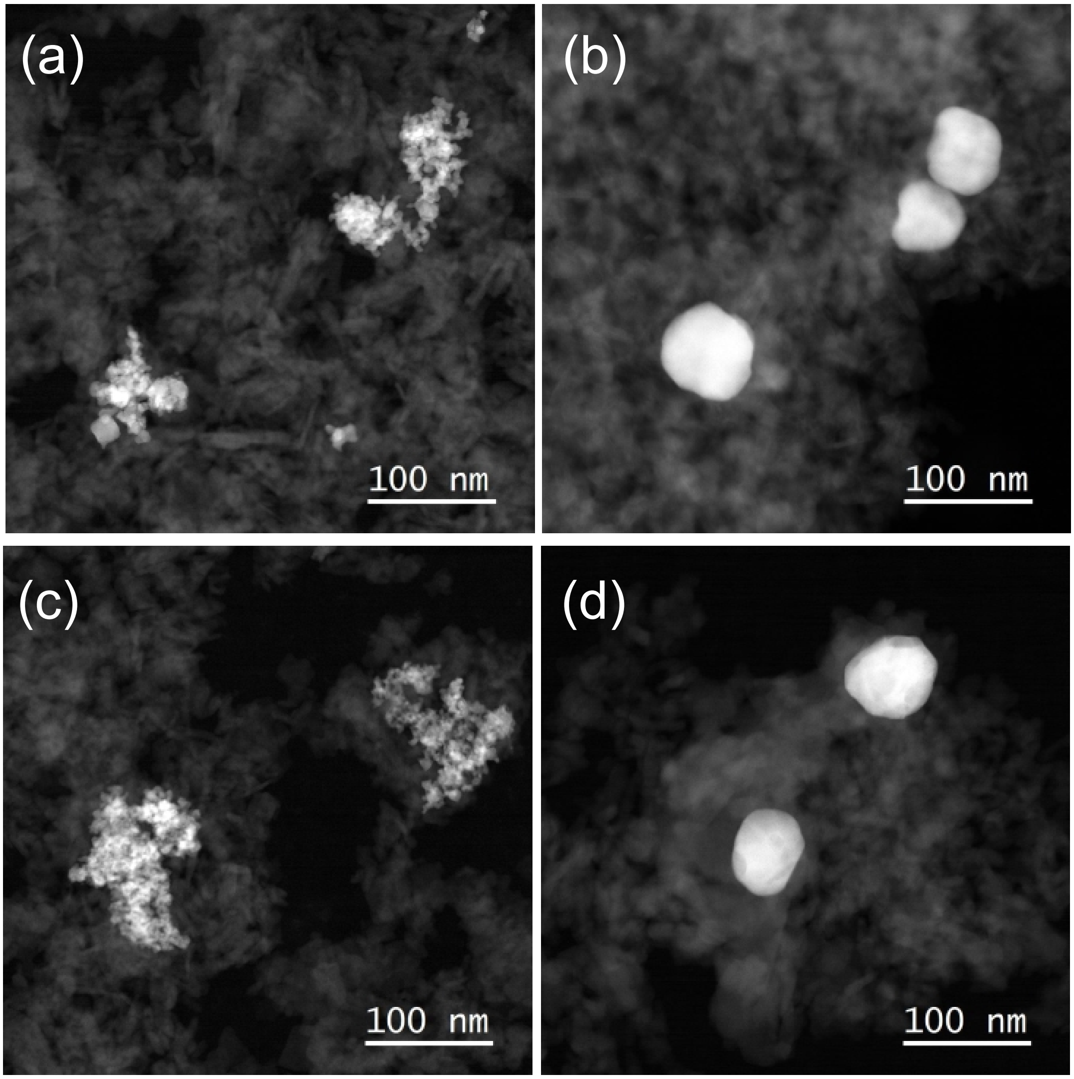

Fig. S2. HAADF-STEM images of (a, b) hts0218 and (c, d) hts0025. Panels (a) and (c) correspond to the states before aging, while (b) and (d) correspond to the states after aging.

(1) : hts0025

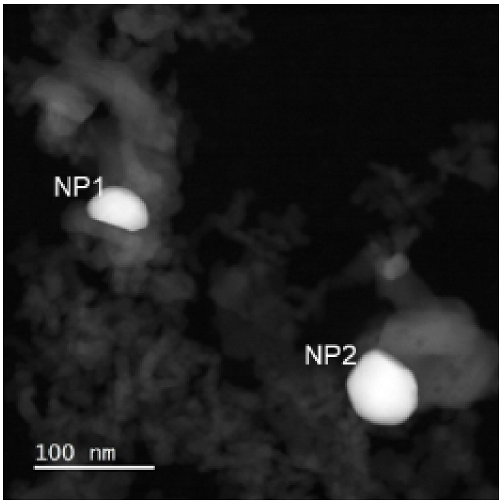

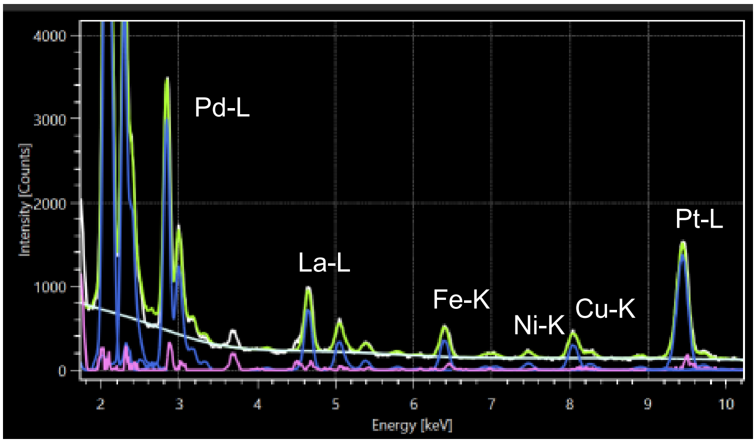

at.%	Fe	Ni	Cu	Pd	Pt
nominal	19	17	11	27	27
entire	7.8	2.0	7.8	39.0	43.5
NP1	4.0	0.9	10.6	40.3	44.2
NP2	1.4	n.d.	3.7	44.3	50.6

(2) : hts0168

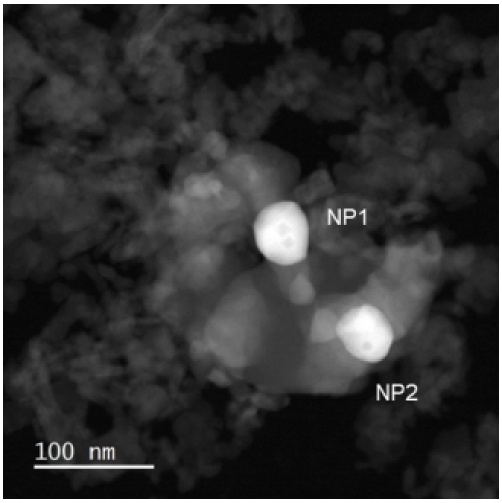

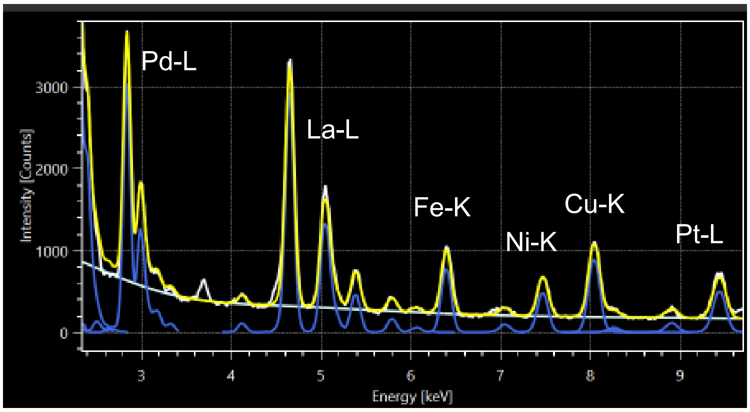

at.%	Fe	Ni	Cu	Pd	Pt
nominal	19	17	23	24	18
entire	15	10.8	21.5	36.6	15.5
NP1	4.0	1.8	15.7	55.2	23.3
NP2	8.6	4.0	15.1	50.6	21.8

Fig. S3 Compositional analysis of (1) hts0025 and (2) hts0168 (with respect to Fe, Ni, Cu, Pd, Pt). (Left) HAADF-STEM image showing two metallic nanoparticles, NP1 and NP2. (Upper right) EDS spectra acquired from the nanoparticles. (Lower right) Table summarizing the nominal compositions of the nanoparticles, along with the EDS analysis results for the entire field of view (entire), NP1, and NP2, respectively. “n.d.” represents “not detected”. The deviation between nominal and locally measured compositions reflects intrinsic elemental segregation during reduction and thermal ageing, rather than experimental uncertainty, which is characteristic of multi-element alloy catalysts.

(1) : hts0025

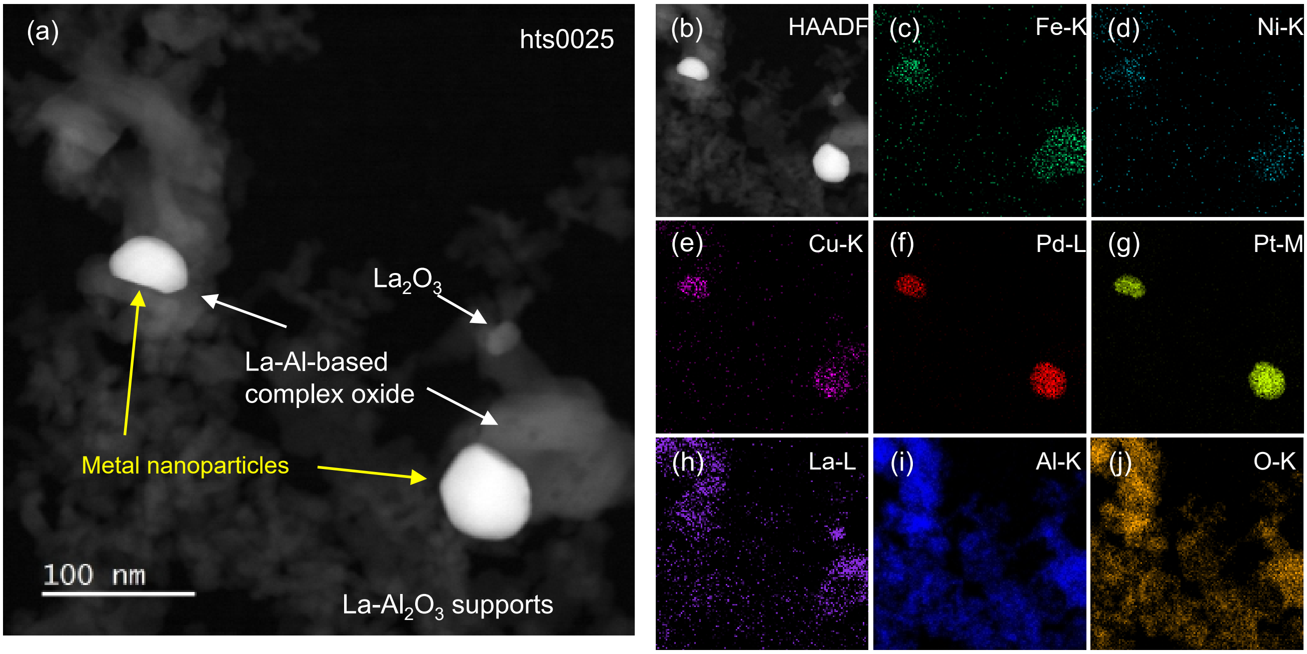

(2) : hts0168

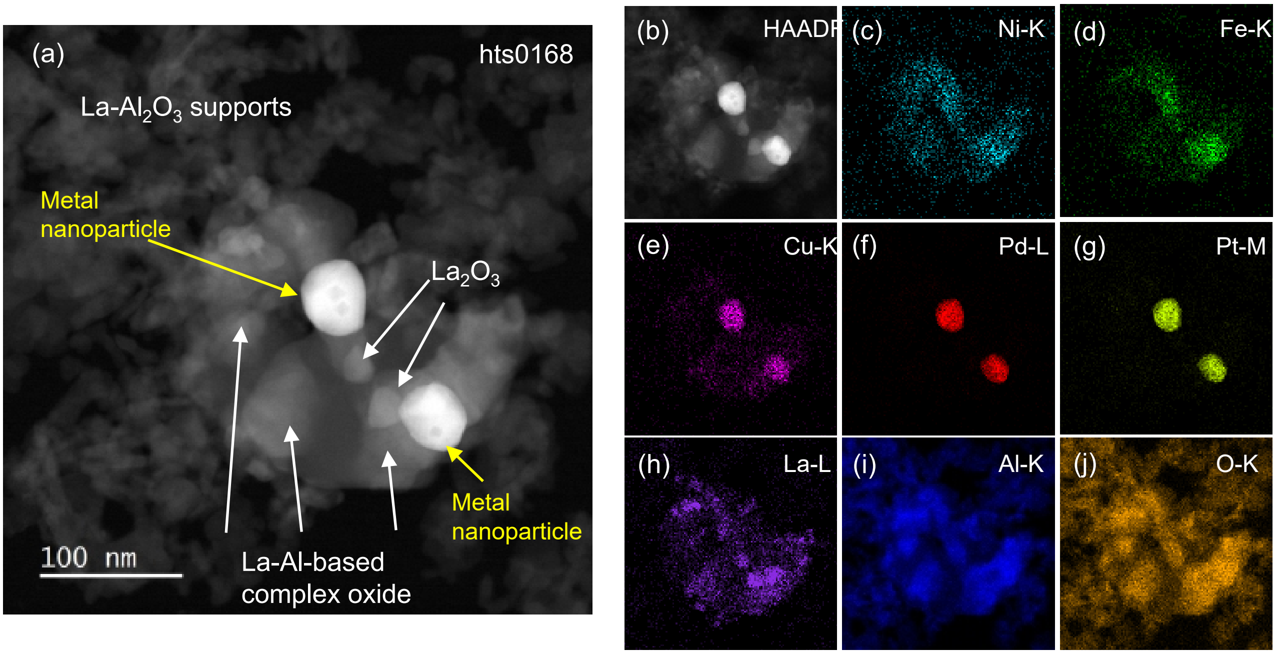

Fig. S4 Electron microscopy analysis of (1) hts0025 and (2) hts0168. (a) HAADF-STEM image showing the morphology of the catalyst. (b) HAADF-STEM image of the same field of view as in (a), to which EDS elemental mapping was applied. (c–i) EDS elemental maps of Fe, Ni, Cu, Pd, Pt, La, Al, and O, respectively.

(a)

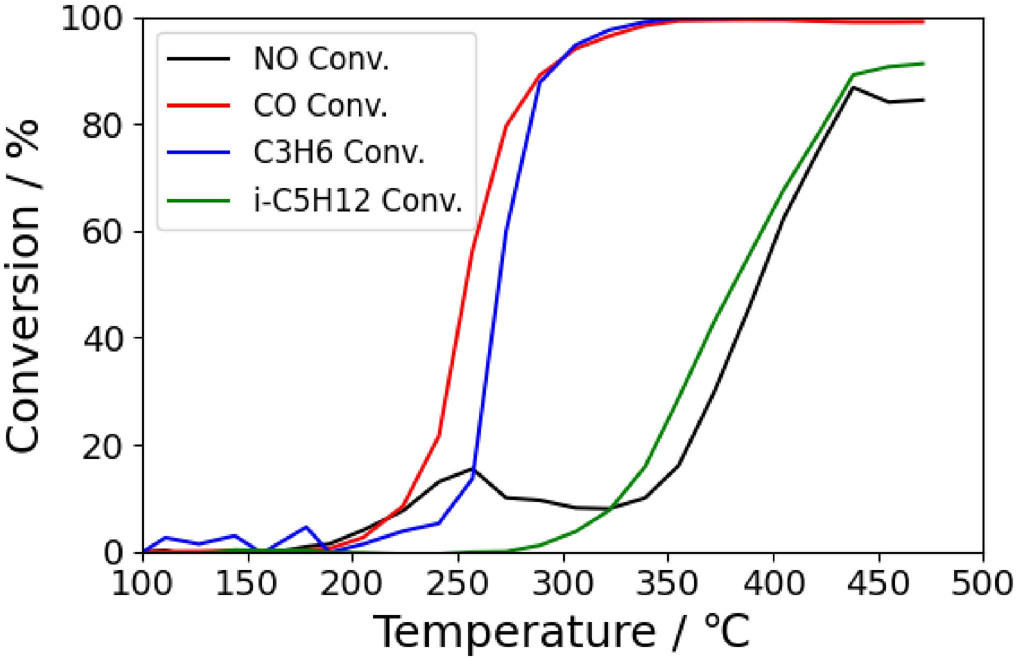

(b)

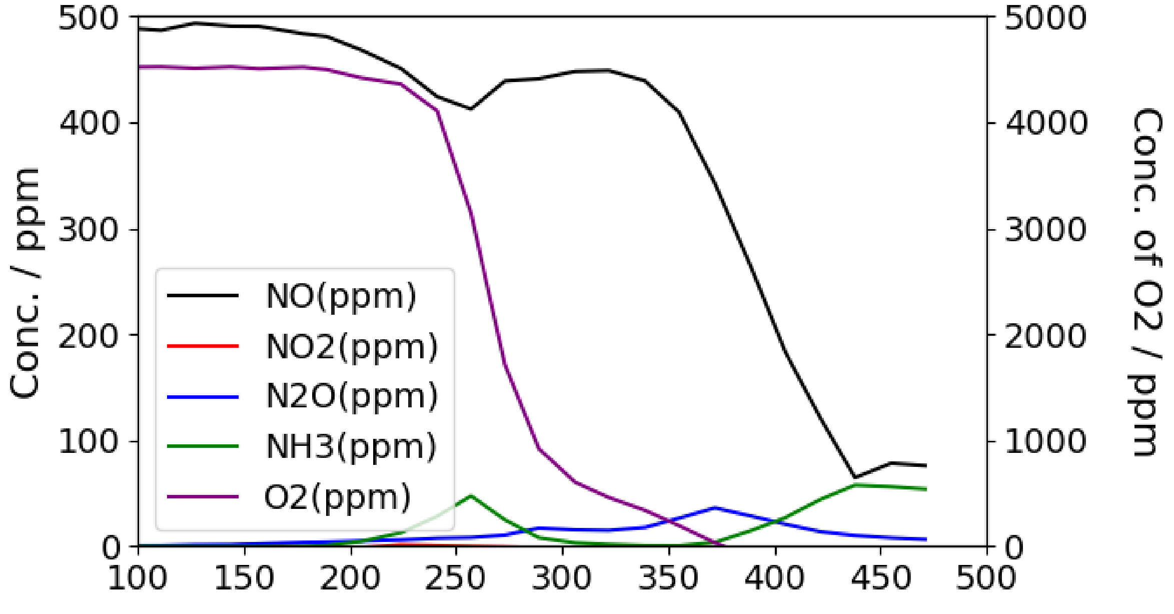

Fig. S5 (a) Light-off curves and (b) concentration of out-gas of 1wt% Pd/Al_2O_3 after aging.

(a)

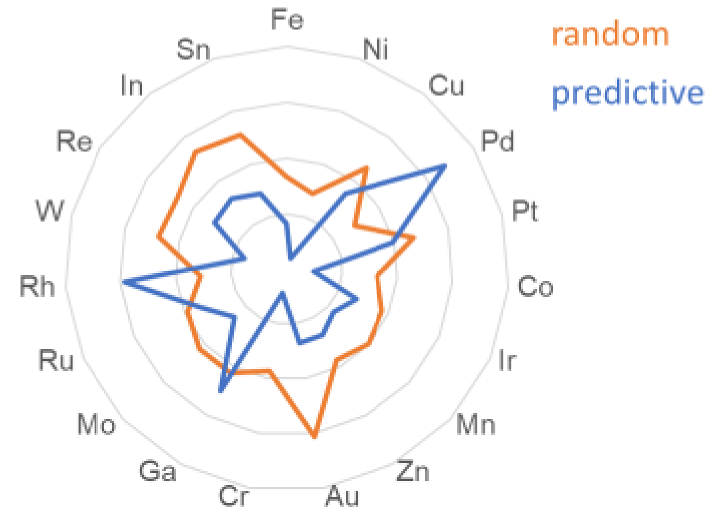

(b)

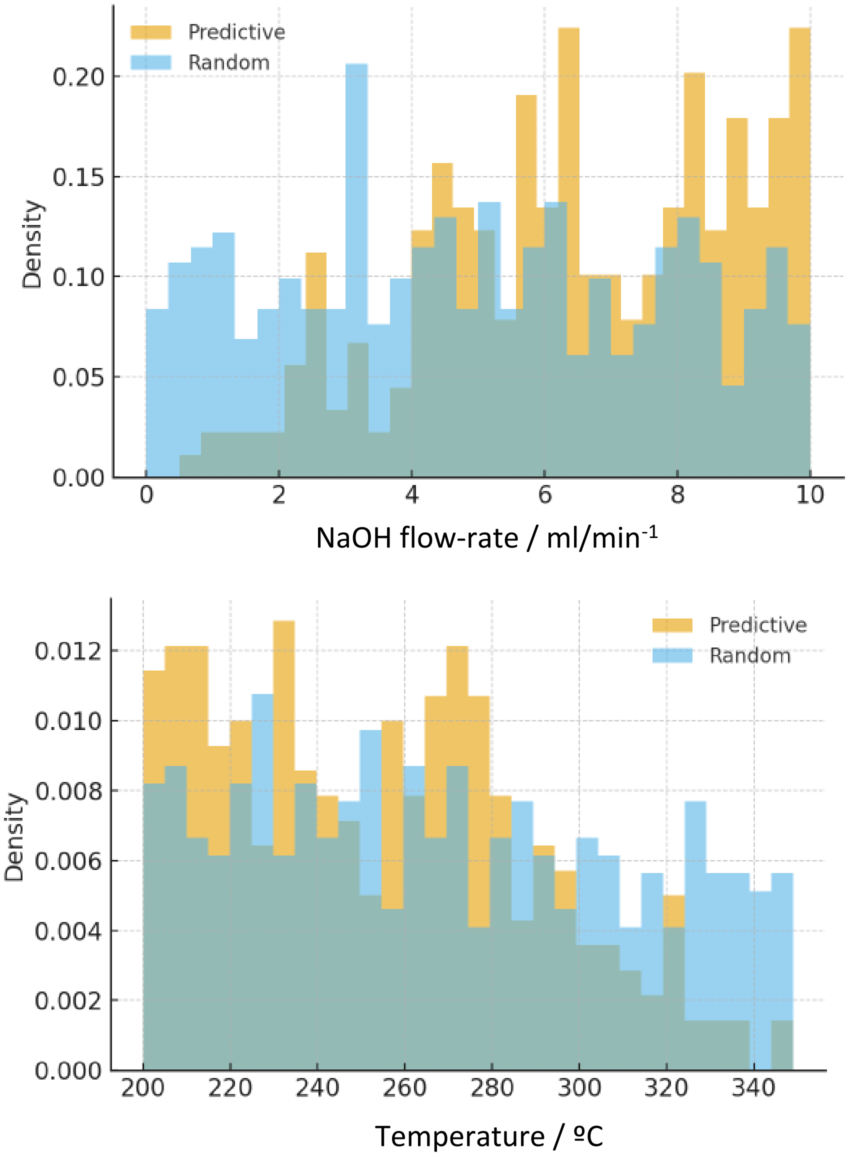

Fig. S6 Exploration behavior in random and predictive high-throughput searches
(a) Elemental sampling bias in random vs predictive exploration, highlighting enrichment in Pd, Rh, and Ga. (b) Shift in synthesis parameters toward higher alkali dosage and lower temperature under predictive conditions.

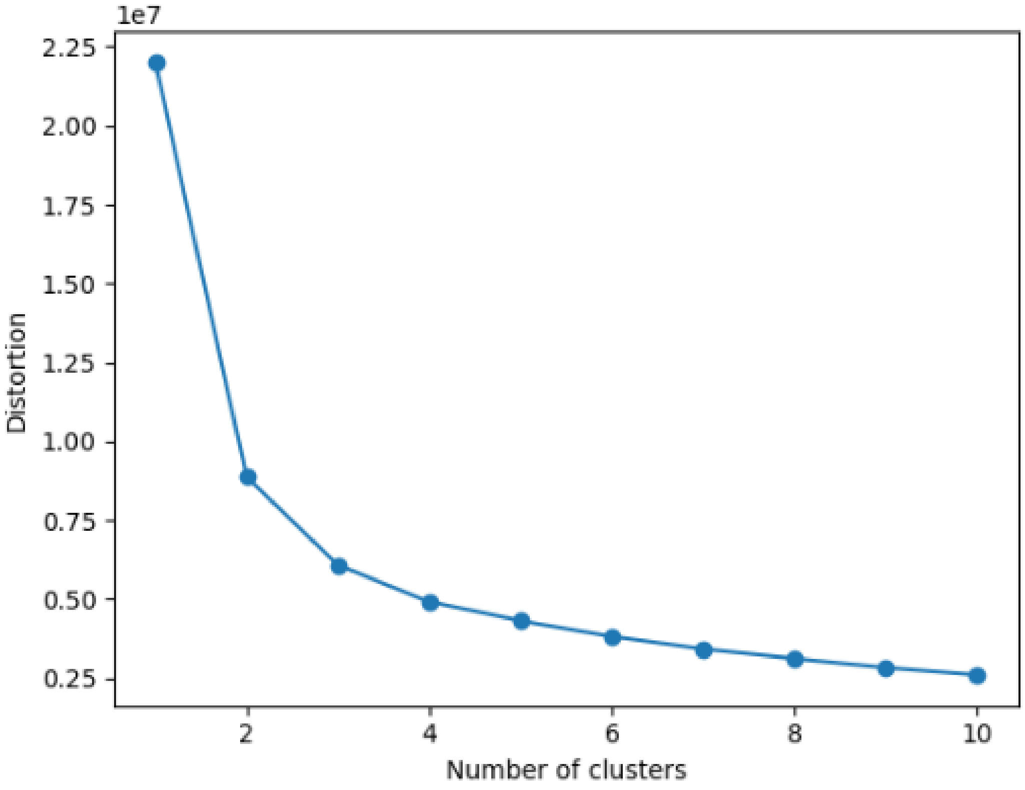

Fig. S7 Elbow plot illustrating the relationship between distortion and the number of clusters of 1493 activity profiles.

Table S2 Top 15 multi-element alloy catalysts ranked by post-ageing total conversion (150–450 °C).

The table lists the rank, sample ID, constituent elements, nominal compositions (at%), total conversion integrated over the temperature range of 150–450 °C after ageing, and the relative total conversion expressed as a fold improvement over the Pd benchmark (×Pd). The relative total conversion (×Pd) is defined as the ratio of the post-ageing total conversion of each catalyst to that of the Pd reference measured under identical evaluation conditions.

Rank	ID	element					Nominal composition (at%)					Relative total conversion vs Pd (×Pd)
1	hts1321	Ga	Rh	Pd	Ir	Pt	11	64	11	7	7	**1.62**
2	hts0902	Mn	Co	Rh	Pd	Pt	4	21	17	4	55	**1.62**
3	hts1324	Ga	Rh	Pd	Re	Ir	19	62	15	2	1	**1.61**
4	hts1544	Ga	Rh	Pd	Ir	Pt	10	56	25	2	6	**1.61**
5	hts1431	Cr	Ru	Rh	Pd	Ir	6	4	79	7	4	**1.60**
6	hts1390	Mn	Ga	Ru	Rh	Pd	6	71	4	14	5	**1.60**
7	hts1293	Ga	Rh	Pd	Re	Pt	28	43	15	3	12	**1.60**
8	hts1532	Ga	Rh	Pd	Ir	Pt	6	71	7	6	11	**1.60**
9	hts1543	Ga	Rh	Pd	Re	Pt	2	83	3	3	9	**1.59**
10	hts1304	Ga	Rh	Pd	W	Pt	19	38	40	2	1	**1.59**
11	hts1514	Mo	Ru	Rh	Pd	Re	5	4	76	9	7	**1.59**
12	hts1374	Mn	Zn	Rh	Pd	Pt	12	6	52	17	12	**1.59**
13	hts1363	Mn	Fe	Rh	Pd	Re	10	3	77	6	4	**1.58**
14	hts1531	Mo	Ru	Rh	Pd	W	8	9	62	13	7	**1.56**
15	hts1343	Ga	Rh	Pd	Re	Pt	21	61	10	5	3	**1.56**
